# Unreported SARS-CoV-2 Home Testing and Test Positivity

**DOI:** 10.1001/jamanetworkopen.2022.52684

**Published:** 2023-01-25

**Authors:** Soo Park, Gregory M. Marcus, Jeffrey E. Olgin, Thomas Carton, Rita Hamad, Mark J. Pletcher

**Affiliations:** 1 Department of Epidemiology and Biostatistics, University of California, San Francisco; 2 Division of Cardiology, Department of Medicine, University of California, San Francisco; 3 Louisiana Public Health Institute, New Orleans; 4 Phillip R. Lee Institute for Health Policy Studies, University of California, San Francisco; 5 Department of Family and Community Medicine, University of California, San Francisco; 6 Division of General Internal Medicine, Department of Medicine, University of California, San Francisco

## Abstract

This cohort study examines time trends in officially reported SARS-CoV-2 case counts and unreported home test positivity.

## Introduction

Timely SARS-CoV-2 testing is critical to reducing transmission. Throughout the COVID-19 pandemic, COVID-19 test sites have been required to report SARS-CoV-2 test results to local or state public health departments,^1^ and these data are used for detecting new surges of transmission. With increasing availability of home antigen tests, however, it is unclear how to interpret time trends in officially reported case counts and test positivity.

## Methods

The COVID-19 Citizen Science Study was approved by the institutional review board at the University of California, San Francisco, and was launched in March 2020 to gather patient-reported data about the COVID-19 pandemic.^2^ This cohort study followed the Strengthening the Reporting of Observational Studies in Epidemiology (STROBE) reporting guideline for cohort studies. Participants were invited by word of mouth or social media or from our recruitment partners via email, telephone, or patient portal message and then provided informed consent and baseline demographic information. Race and ethnicity were self-reported by the participants and were analyzed in this study to understand differences in unreported test frequency and test positivity. Each week, we asked participants about recent COVID-19 testing and test results. In March 2022, a question was added to distinguish tests conducted with a “Fully at-home test kit, with my own sample collection and reading of my own results” vs tests where “a healthcare provider collected my sample” or that were “sent to a clinical lab.”

To compare with SARS-CoV-2 testing reported nationally, we downloaded data from the Johns Hopkins Coronavirus Resource Center^3^ on August 15, 2022, and plotted smoothed 7-day moving averages of total daily tests and test positivity. With COVID-19 Citizen Science Study data, we fit mixed logistic models with a random intercept for each participant and fixed effects for time and demographic characteristics. We analyzed data using Stata statistical software version 17 (StataCorp), using 2-sided tests with α = .05 for hypothesis testing.

## Results

Of 102 591 US participants enrolled, 18 642 (18%) reported completing at least 1 SARS-CoV-2 test from March 16, 2022, to August 15, 2022; 18 546 participants also had complete demographic information. Most were female (12 568 participants [67.8%]) and non-Hispanic White (15 231 participants [82.1%]), with a mean (SD) age of 55 (14) years. During this time period, the proportion of SARS-CoV-2 testing conducted at home increased from approximately 60% to more than 80% (Figure) (*P* < .001 for time trend). The percentage test positivity on home tests was similar to officially reported tests^3^ through June, but then started to diverge with lower positivity in home tests (*P* < .001 for interaction of official test positivity and time). Female, non-Hispanic White, younger, and higher social status participants were more likely to test at home. Male participants and young adults were more likely to test positive on home tests (Table).

**Figure.**
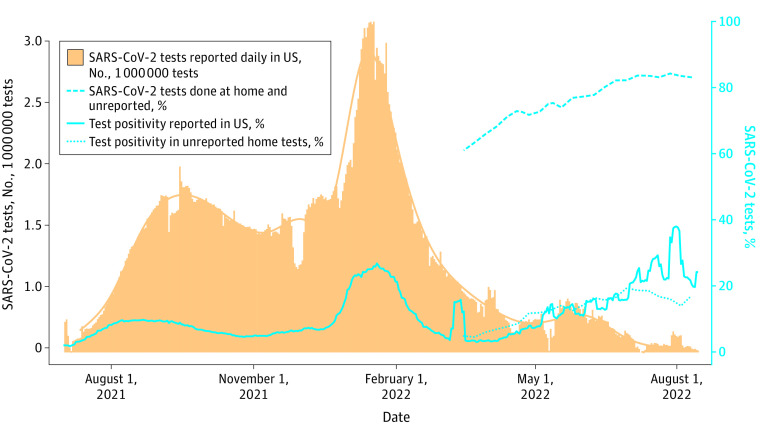
SARS-CoV-2 Testing, Test Type, and Test Positivity Trends Over Time The solid lines represent total SARS-CoV-2 tests reported daily (with smoothed trend line) and percentage 7-day test positivity from July 1, 2021, to August 15, 2022, in the US, extracted from the Johns Hopkins Coronavirus Resource Center website.^3^ The dashed lines represent data on home testing from the COVID-19 Citizen Science Study.

**Table.** Unreported SARS-CoV-2 Testing and Test Positivity From March 16, 2022, to August 15, 2022, by Participant Characteristic

Characteristic	Total SARS-CoV-2 tests reported, No. (% of sample)^b^	Tests conducted at home and unreported^a^		Home test is positive	
		No. (%)^c^	Adjusted OR (95% CI)^d^	No. (%)^e^	Adjusted OR (95% CI)^d^
Overall	84 027 (100)	64 616 (76.9)	NA	8869 (13.7)	NA
Sex					
Male	25 677 (30.6)	19 224 (74.9)	0.79 (0.72-0.86)	3124 (16.3)	1.63 (1.45-1.83)
Female	58 177 (69.2)	45 266 (77.8)	1 [Reference]	5739 (12.7)	1 [Reference]
Other or unknown	173 (0.2)	126 (72.8)	0.71 (0.25-2.00)	6 (4.8)	0.31 (0.06-1.59)
Race and ethnicity					
African American or Black, non-Hispanic	1551 (1.9)	1042 (67.2)	0.36 (0.28-0.47)	135 (13.0)	1.04 (0.69-1.56)
Asian, non-Hispanic	4388 (5.2)	3310 (75.4)	0.79 (0.66-0.94)	402 (12.1)	0.68 (0.52-0.87)
Hispanic or Latinx, any race	5437 (6.5)	3938 (72.4)	0.65 (0.56-0.76)	567 (14.4)	1.02 (0.82-1.26)
White, non-Hispanic	68 675 (81.7)	53 240 (77.5)	1 [Reference]	7335 (13.8)	1 [Reference]
Other or unknown^f^	3976 (4.7)	3086 (77.6)	0.88 (0.73-1.06)	430 (13.9)	1.03 (0.80-1.32)
Age, y					
18-49	34 150 (40.7)	26 715 (78.2)	1 [Reference]	3885 (14.5)	1 [Reference]
50-64	28 308 (33.7)	21 766 (76.9)	0.85 (0.78-0.94)	2983 (13.7)	0.69 (0.61-0.78)
≥65	21 555 (25.7)	16 124 (74.8)	0.61 (0.56-0.68)	2000 (12.4)	0.50 (0.43-0.57)
Subjective social status^g^					
1-6	23 371 (27.8)	17 256 (73.8)	0.71 (0.65-0.78)	2459 (14.3)	1.05 (0.93-1.20)
7	23 265 (27.7)	17 774 (76.4)	0.88 (0.80-0.97)	2307 (13.0)	0.96 (0.85-1.09)
8-10	37 386 (44.5)	29 581 (79.1)	1 [Reference]	4103 (13.9)	1 [Reference]

Abbreviations: NA, not applicable; OR, odds ratio.

^a^ Refers to home tests that were not necessarily reported to an authority.

^b^ Partial responses (n = 2) with no test result or type of testing performed and participants without demographic information (n = 91) were excluded from analyses.

^c^ Percentage is calculated as total home tests divided by total tests.

^d^ Multilevel mixed-effects logistic regression was used to calculate adjusted ORs and 95% CIs.

^e^ Percentage is calculated as home tests that are positive divided by total home tests.

^f^ Includes Native Hawaiian or Pacific Islander, American Indian or Alaska Native, some other race, do not know, and multiracial.

^g^ Refers to MacArthur Subjective Social Status Scale, a measure of perceived social standing on a 1 to 10 scale, with 1 denoting worst off and 10 denoting best off.

## Discussion

In this cohort study, we found home testing to be increasingly common through spring and into summer 2022, most recently comprising more than 80% of all SARS-CoV-2 testing reported. Home test positivity appears to track closely with national data from reported tests, but these trends are starting to diverge. Home testing patterns differ by demographic subgroup, as previously shown,^4^ perhaps because of differential COVID-19 worry or availability and cost of test kits. Our study has limitations. Home testing may be conducted repeatedly during an illness episode, may be less common in the US population than in our engaged participants, and may sometimes be officially reported despite being conducted at home (eg, via employers), all of which could bias our estimates of test positivity and the proportion of tests that are unreported.

Our findings confirm common wisdom^5,6^ that official COVID-19 case counts increasingly underestimate the number of people who test positive and vastly underestimate the number of true infections. The percentage test positivity in officially reported tests appears to reflect home test positivity, though these trends may be diverging.

## Notes

Supplement 1.
COVID-19 Citizen Science Study Consortium Members

Supplement 2.
Data Sharing Statement
