# Supplementary material for: Unreported SARS-CoV-2 Home Testing and Test Positivity
Source: JAMA Netw Open. 2023 Jan 25;6(1):e2252684. doi: 10.1001/jamanetworkopen.2022.52684 (PMC10187483; doi:10.1001/jamanetworkopen.2022.52684)
Supplement: Supplement 1. — COVID-19 Citizen Science Study Consortium Members [file jamanetwopen-e2252684-s001.pdf]

Supplemental Online Content: Nonauthor Collaborators
*First name, last name, and suffix (if applicable) are required and will appear in PubMed.

*Group Name(s): The COVID-19 Citizen Science Study Consortium							
***First Name and Middle Initial(s)**	***Last Name**	***Suffix (eg, Jr, III)**	Academic Degrees	Institution	Location (city, state/province, country)	Role or Contribution, eg, chair, principal investigator	Group (if more than 1 Group listed in the byline) and/or Subgroup (eg, Steering Committee)
Rasha	Khatib		PhD, MHS	Advocate Aurora Health	Downers Grove, IL	Co-Investigator	Recruitment
Carmen R.	Isasi		MD, PhD	Albert Einstein College of Medicine	Bronx, NY	Co-Investigator	Steering Committee
Paul	Meissner		MSPH	Albert Einstein College of Medicine	Bronx, NY	Site Informatics	Policy Core
Heather	Kitzman		PhD	Baylor Scott & White Health	Dallas, TX	Co-Investigator	Steering Committee
Djeneba Audrey	Djibo		PhD	CVS Health	Blue Bell, PA	Co-Investigator	Vulnerable Population Core
Emily	O'Brien		PhD	Duke University School of Medicine	Durham, NC	Co-Investigator	Steering Committee
Pelin	Ozluk		PhD	HealthCore, Inc.	Wilmington, DE	Co-Investigator	Policy Core
Gosia	Sylwestrzak		MA	HealthCore, Inc.	Wilmington, DE	Co-Investigator	Policy Core
Alan	Kaul		PharmD, M	Practice Research Network (PRACnet	Sharon, MA	Co-Investigator	Policy Core
Vinit	Nair		B.Pharm, M	Practice Research Network (PRACnet	Sharon, MA	Co-Investigator	Policy Core
Tom	Carton		PhD	Louisiana Public Health Institute	New Orleans, LA	Dual Principal Investiga	Steering Committee
Erica	Johnson		MPH	Louisiana Public Health Institute	New Orleans, LA	Project Manager	Steering Committee
Janna	Garcia Torres		MA	New York University Grossman Schoo	New York, NY	Project Coordinator	Recruitment
Claudia	Pulgarin		MS	New York University Grossman Schoo	New York, NY	Site Informatics	Data Core
Natasha	Williams		EdD, MPH	New York University Grossman Schoo	New York, NY	Co-Investigator	Steering Committee
Julie	Castex		MSN	Ochsner Clinic Foundation	New Orleans, LA	Co-Investigator	Steering Committee
Amy	Feehan		PhD	Ochsner Clinic Foundation	New Orleans, LA	Co-Investigator	Steering Committee
Kristen	Azar		MSN, MPH	Sutter Health	Sacramento, CA	Co-Investigator	Steering Committee
Alice	Pressman		PhD	Sutter Health	Sacramento, CA	Co-Investigator	Policy Core
Sylvia	Sudat		PhD	Sutter Health	Sacramento, CA	Co-Investigator	Data Core
Matthew	Brandner		BA	University of California, San Francisco	San Francisco, CA	Clinical Research Coord	Recruitment
Madelaine F.	Modrow		MPH	University of California, San Francisco	San Francisco, CA	Project Director	Steering Committee
Rita	Hamad		MD, PhD	University of California, San Francisco	San Francisco, CA	Co-Investigator	Steering Committee
John	Kornak		PhD	University of California, San Francisco	San Francisco, CA	Statistician	Data Core
Greg	Marcus		MD, MAS	University of California, San Francisco	San Francisco, CA	Co-Principal Investigato	Steering Committee
Jeff	Martin		MD, MPH	University of California, San Francisco	San Francisco, CA	Co-Investigator	Steering Committee
Jeffrey	Olgin		MD	University of California, San Francisco	San Francisco, CA	Co-Principal Investigato	Steering Committee
Jaime	Orozco		BA	University of California, San Francisco	San Francisco, CA	Clinical Research Coord	Vulnerable Population Core
Soo	Park		BA	University of California, San Francisco	San Francisco, CA	Data Manager	Policy Core
Noah	Peyser		PhD	University of California, San Francisco	San Francisco, CA	Eureka Platform Direct	Eureka Platform
Mark J.	Pletcher		MD, MPH	University of California, San Francisco	San Francisco, CA	Dual Principal Investiga	Steering Committee

Supplemental Online Content: Nonauthor Collaborators
*First name, last name, and suffix (if applicable) are required and will appear in PubMed.

***First Name and Middle Initial(s)**	***Last Name**	***Suffix (eg, Jr, III)**	Academic Degrees	Institution	Location (city, state/province, country)	Role or Contribution, eg, chair, principal investigator	Group (if more than 1 Group listed in the byline) and/or Subgroup (eg, Steering Committee)	
Sara	Knight		PhD	University of Utah	Salt Lake City, UT	Co-Investigator	Steering Committee	
Ana	Sanchez-Birkhead		PhD	University of Utah	Salt Lake City, UT	Co-Investigator	Vulnerable Population Core	
Mark	Weiner		MD	Weill Cornell Medicine	New York, NY	Co-Investigator	Steering Committee	
