# Supplementary material for: Unreported SARS-CoV-2 Home Testing and Test Positivity
Source: JAMA Netw Open. 2023 Jan 25;6(1):e2252684. doi: 10.1001/jamanetworkopen.2022.52684 (PMC10187483; doi:10.1001/jamanetworkopen.2022.52684)
Supplement: Supplement 2. — Data Sharing Statement [file jamanetwopen-e2252684-s002.pdf]

Data Sharing Statement

Park. Unreported SARS-CoV-2 Home Testing and Test Positivity. *JAMA Netw Open*.
Published January 25, 2023. doi:10.1001/jamanetworkopen.2022.52684

Data
Data available: Yes
Data types: Deidentified participant data, Data dictionary
How to access data: Upon request from the authors
When available: With publication

Supporting Documents
Document types: Statistical/analytic code
How to access documents: Upon request from the authors
When available: With publication

Additional Information
Who can access the data: Researchers whose proposed use of the data has been approved
Types of analyses: For a specified purpose
Mechanisms of data availability: With a signed data access agreement
